# To be or not to be active – a matter of attitudes and social support? Women’s perceptions of physical activity five years after Roux-en-Y Gastric Bypass surgery

**DOI:** 10.1080/17482631.2019.1612704

**Published:** 2019-05-10

**Authors:** Sofie Possmark, Daniel Berglind, Fanny Sellberg, Ata Ghaderi, Margareta Persson

**Affiliations:** a Department of Public Health Sciences, Karolinska Institutet, K9, Social Medicin, Stockholm, Sweden; b Department of Clinical Neuroscience, Karolinska Institutet, Stockholm, Sweden; c Department of Nursing, Umeå University, Umeå, Sweden

**Keywords:** Roux-en-Y gastric bypass, bariatric surgery, physical activity, attitudes, social support, interviews, qualitative, grounded theory

## Abstract

**Purpose**: Despite positive health advantages of post-surgery physical activity (PA) for bariatric surgery patients, the majority is not sufficiently physically active. The aim was to explore women’s perceptions and experiences concerning PA five years after Roux-en-Y Gastric Bypass (RYGB) surgery.

**Methods**: Eleven women were interviewed five years post-surgery. Grounded Theory approach was applied.

**Results**: The core-category *“Attitudes and surrounding environment influence activity levels”* includes three attitudes towards PA: *“Positive attitudes”, “Shifting attitudes”* and *“Negative attitudes”*. Participants with a positive attitude were regularly physically active, felt supported and proud of their achievements. Contrary, participants with a negative attitude didn’t prioritize PA, didn’t feel supported and saw no need or benefit of PA. Some participants revealed an on-off behaviour, hovering between the attitudes of vigorous PA and sedentary lifestyle, without sustainable balance. The majority mostly viewed PA as a mean to lose weight.

**Conclusion**: The level of perceived post-surgery PA was related to the participants’ attitudes towards PA and whether or not they had a supportive environment. These findings might explain why bariatric surgery patients often fail to be sufficiently active post-surgery, and highlight the need for prolonged support and motivational interventions to promote sustainable PA post-bariatric surgery.

## 1. Introduction

Obesity has become a worldwide problem where 11% of the men and 15% of the women are obese (Collaboration NRF, 2016). In Sweden, half of the population (51%) is either overweight or obese, and the prevalence of obesity is approximately 15% for both men and women (Folkhälsomyndigheten, 2018). The most effective strategy for sustainable weight loss and improvements in obesity-related diseases has proven to be bariatric surgery (Adams et al., 2017; Colquitt, Pickett, Loveman, & Frampton, 2014), such as Roux-en-Y Gastric Bypass (RYGB) surgery.

Physical activity (PA) can help bariatric surgery patients to maintain their weight loss after surgery (Wefers et al., 2016), especially participating in moderate-to-vigorous intensity PA (MVPA) can be an important part in regulating body weight and contribute to improve surgical outcomes after bariatric surgery (Jacobi, Ciangura, Couet, & Oppert, 2011; Wc & Ds, 2013). However, several studies have shown that bariatric surgery patients often fail to reach the recommended levels of at least 150 minutes of MVPA per week (WHO, 2011), when objective measures of PA have been used (Bergh, Kvalem, Mala, Hansen, & Sniehotta, 2017; Berglind et al., 2015, 2016; Bond et al., 2010; Sellberg, Willmer, Tynelius, & Berglind, 2017). A systematic review of objectively measured PA after bariatric surgery shows little change in PA up to one-year post-surgery (Herring et al., 2016). A cohort following women up to four years post-RYGB-surgery shows no significant differences in objectively measured PA-levels compared to three months pre-surgery. Also, the prevalence of women who reach the recommended guidelines before and four years after surgery is low (Sellberg et al., 2017). In addition, when comparing self-reported PA to objectively measured PA within the same individual, patients undergoing RYGB tend to increase their self-reported PA-levels more than objectively measured PA after surgery, compared to pre-surgery (Berglind et al., 2016).

Qualitative interview studies about post-surgery PA have mostly been focused on barriers or facilitators to PA engagement after bariatric surgery. For example, described barriers to PA after bariatric surgery are discomfort due to excess skin or other weight-related physical difficulties (Dikareva, Harvey, Cicchillitti, Bartlett, & Andersen, 2016; Stenmark Tullberg, Fagevik Olsén, Shams, & Wiklund, 2017; Zabatiero et al., 2017), lack of social support (Dikareva et al., 2016; Stenmark Tullberg et al., 2017; Zabatiero et al., 2017), lack of time (Benson-Davies, Davies, & Kattelmann, 2013; Dikareva et al., 2016; Zabatiero et al., 2017) and lack of motivation (Dikareva et al., 2016; Lier, Aastrom, & Rortveit, 2016; Zabatiero et al., 2017). Weight loss and to be able to move more easily have been shown to be facilitators for being physically active (Dikareva et al., 2016; Zabatiero et al., 2017), as well as having social support in the closest environment (Dikareva et al., 2016; Zabatiero et al., 2017). Women participating in a post-surgery exercise program reveals that a standardized exercise program does not suit everybody. Furthermore, their experiences of the program are related to the perceived extent that the surgery had affected their lives (Groven, Råheim, & Engelsrud, 2015). Even though barriers and facilitators to PA have been explored, no study to our knowledge has qualitatively elucidated the reasons behind bariatric patients’ failure to reach the recommended weekly levels of MVPA after surgery. A question of special interest is why these barriers exist for some patients, while others manage to become more active post-surgery.

In summary, PA is important for patients undergoing bariatric surgery, but most patients do not meet the recommended levels of MVPA. The reasons why this patient group does not meet the recommended levels of MVPA, as well as how they perceive and experience PA over a longer time period post-surgery, are sparsely studied. Therefore, the aim of this study is to qualitatively explore the women’s perceptions and experiences concerning PA five years after RYGB surgery.

## 2. Material and methods

### 2.1 Design

In this qualitative interview study, a Grounded Theory (GT) approach inspired by Corbin and Strauss (Corbin, 2008) was applied. GT is an abductive method as it involves a cyclic process, moving back and forth between the empirical data and the emerging theory. By using this approach, construct and theory emerge through and are grounded in the data (Corbin, 2008; Green, 2009).

### 2.2 Recruitment and data collection

Potential participants for this interview study comprises RYGB-treated women from a previous cohort study recruited three months pre-surgery and followed up to four years post-surgery. Time of surgery was between June 2012 and January 2013. A detailed description of the initial cohort and the subsequent follow-ups are published elsewhere (Berglind et al., 2015, 2016; Sellberg et al., 2017).

All women who participated in the four-year follow-up (n = 38) were contacted by mail in March and April 2017. Two subsequent reminders were sent out approximately four weeks apart, containing the same information as the first letter. When a signed informed consent was returned, the researcher (SP) contacted the participants by telephone to provide additional information if needed and, thereafter, decided time and place for the interview. Fourteen women returned an informed consent, of which two women never responded to phone calls and one woman decided to drop-out due to an up-coming emigration, resulting in 11 eligible participants to interview.

A topic guide based on literature and previous research findings were developed by SP, DB and MP to guide the interviews. The interview guide included three central topics covering various aspects of post-surgery life. PA was one of the topics, and the remaining data will be presented elsewhere. The main questions covering PA are presented in Table I.

**Table I.** Main topics of the interview guide about physical activity (PA).

Main topics asked during interview
- What is PA for you? - How do you perceive yourself when it comes to PA and exercise? - During the last month, how would you describe your PA? - What obstacles do you perceive you have in order to achieve a good (for you) level of PA and/or exercise? - What is your knowledge about PA? If any knowledge, where did you acquire it? - What are your thoughts about exercise and weight loss, compared to exercise and wellbeing? - Do you have any support to be active? - Do you have problems with loose skin after surgery, and if so, have it been bothering you during PA or exercise? - What advice about PA and exercise would you give to someone who were about to undergo a RYGB-surgery?

An emergent design was applied, meaning that the interview guide could be updated between the interviews, adding new topics that needed further investigation, as well as enriching the subsequent interviews (Corbin, 2008). This resulted in minor changes to the interview guide after the preliminary analysis of each interview. The interviews were conducted between April 2017—March 2018. Eight of the interviews were held in the homes of the participants, two interviews were held in the researcher’s (SP) office and one interview was held over the telephone. All locations were chosen by the participants. The lengths of the interviews varied between 36 minutes and 120 minutes. At each interview the women’s height, weight and waist circumference were measured. No height or waist circumference were measured for the woman who participated over the phone. She had earlier that day had her five-year standard follow-up at the hospital where she had had surgery, and the weight reported was measured by a nurse the same morning.

All interviews were audio recorded. Immediately after each interview, SP wrote memos which included reflections and overall impressions from the interview together with ideas and questions that could be considered for the development of the interview guide as well as for the preliminary analysis. If the saturation of data was not established after the 11 interviews, the plan was to recruit more women from the cohort. The last three interviews did not reveal any major new findings, which indicated that the saturation of data was obtained; thus, no further recruitment was needed.

All interviews were conducted by the same researcher (SP), except for the first interview where an experienced qualitative researcher (MP) also participated. SP has a background in public health and at the time of the interviews, she had minor experiences of encountering bariatric surgery patients.

### 2.3 Data analysis

The analysis followed the steps of GT as described by Corbin and Strauss (Corbin, 2008). Data collection and data analysis were a parallel process, where concepts and categories emerged. These emerging concepts were used to deepen the following data collection, in other words, to find new topics to explore and to see if the emerged concepts and categories could be found in the forthcoming interviews. This procedure went on until saturation was reached, and no major new concepts emerged during the data collection. Consequently, an abductive approach was used during data collection and data analysis (Corbin, 2008; Green, 2009).

All interviews were transcribed verbatim by SP, and preliminary analysis and coding were performed after each transcript. All codes responding to the aim of this study were identified, resulting in more than 600 codes. The codes were thereafter compared, and similar codes were assembled to create properties. Categories were developed that contained properties relating to each other. During the comparison of differences and similarities, the core category emerged, covering the most prominent codes that could be traced through the data. Memos were also used to gather and track ideas about the emerging findings. An explanatory model was developed including the core category, categories and its properties, to explain the processes regarding PA after bariatric surgery. The analysis was mainly performed by SP, in continuous collaboration and discussion with the research group throughout the whole process, in order to improve the quality of the analysis and to minimize the risk of invented data, misinterpretation or misunderstanding (Polit, 2016). An overview of the core category, categories and properties can be seen in Table II.

**Table II.** An overview of the categories and properties belonging to the core-category “Attitudes and surrounding environment influence activity levels”.

Core category	Categories	Properties
Attitudes and surrounding environment influence activity levels	Positive attitudes	Positive image of PA
		Challenge myself to improve
		To feel healthier and happier
		Social support to be active
		Develop strategies to remain active
	Shifting attitudes	Constantly on and off, with some support
	Negative attitudes	Negative image of PA
		Priorities and wishes
		No social support to be active
		Physical limitations for PA
		Exercise is only equal to dieting

### 2.4 Ethical considerations

The research project was approved by the Ethical Review Board in Stockholm (No 2009/1472–31/3 and 2016/836–32). All participants received written and verbal information about the study and signed an informed consent prior to the interviews. Interviews were digitally recorded with permission from the participants. Participants’ anonymity was maintained during the whole process of the analysis and reporting. An important aspect to consider is that people with overweight and obesity often come across stigmatization and discrimination (Puhl & Heuer, 2010). Thus, a respectful and non-judgmental approach during the interviews was important irrespective of participants’ present lifestyle and activity level.

## 3. Results

### 3.1 Characteristics of included participants

In total, interviews were conducted about 5.3 years (SD 0.6) after surgery. At the time of the interviews, the mean age of the participants was 46.2 years (SD 5.8) and the mean BMI was 26.9 kg/m^2^ (SD 3.2). See Table III for descriptive characteristics of the participants. Most women worked full-time, had children still living at home and cohabited with a partner. Seven out of 11 participants lived with the same partner as before their surgery. Three women had certain circumstances that affected and/or impaired their lives in different ways, such as long-term diseases or having a child with special needs.

**Table III.** Characteristics of participants.

Participant	Age (years)	Time since surgery (years)	BMI before surgery (kg/m^2^)	BMI at interview (kg/m^2^)	Level of education
1	48.5	4.4	39.8	25.4	<3 years university
2	46.0	4.5	44.7	28.0	High school
3	54.9	4.7	41.0	29.1	≥3 years university
4	45.1	5.1	36.2	21.0	High school
5	35.9	5.4	48.6	28.6	High school
6	48.2	5.6	39.4	25.9	≥3 years university
7	45.0	5.2	37.4	31.9	<3 years university
8	38.1	5.4	37.1	23.6	High school
9	56.0	5.1	37.5	25.8	High school
10	43.4	6.8	39.3	31.7	<3 years university
11	46.9	5.9	33.3	24.7	≥3 years university

### 3.2 “Attitudes and surrounding environment influence activity levels”

**Figure 1.**
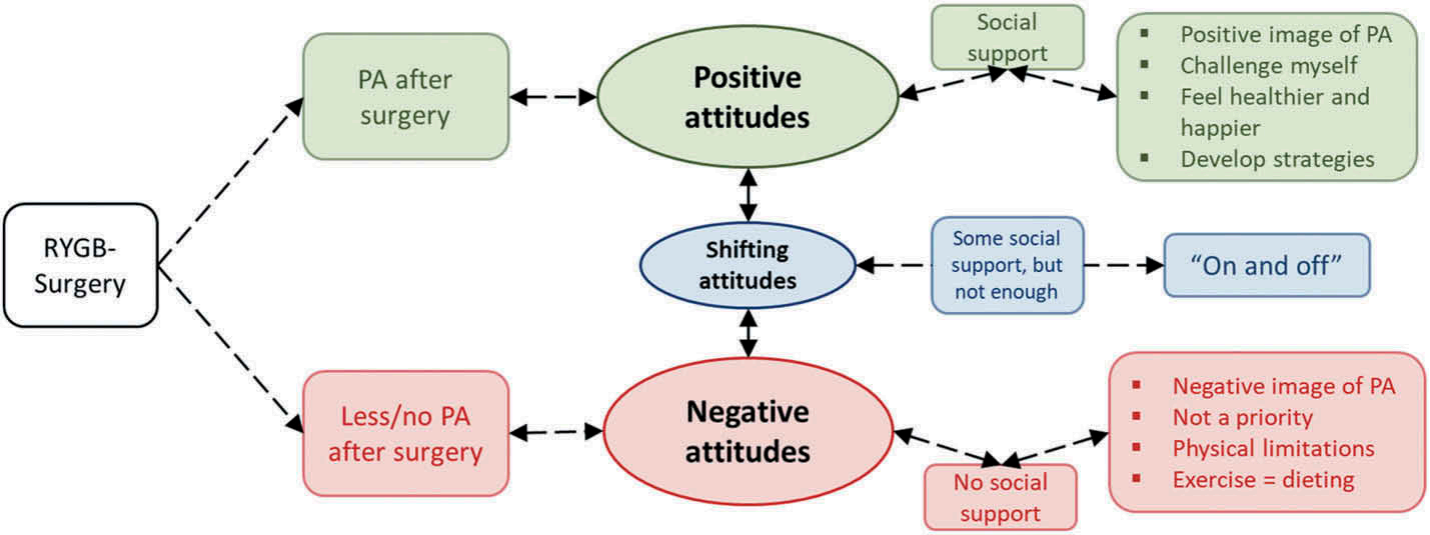
Explanatory model explaining the core category “Attitudes and surrounding environment influence activity levels”.

The core category *“Attitudes and surrounding environment influence activity levels”* together with three categories and corresponding properties are visualized in an explanatory model (Figure 1). The model highlights participants’ attitudes towards PA, which may help explain why regular PA and reaching the recommendations of MVPA are not obtained post-surgery. Some participants revealed a *“Positive attitude”* and had strategies to keep up their PA on a regular basis. Others described a *“Shifting attitude”* where they eagerly started up PA, but never managed to make PA a sustainable habit. Furthermore, a *“Negative attitude”* also emerged among participants who did not see any need of PA as they had lost weight anyway. The findings also emphasized the need for a supportive environment as to maintain PA habits. A comprehensive presentation of the findings comprising the categories and their corresponding properties, with some quotations from the interviews, follow below. The classification into different categories is based on the participants’ own statements on whether or not they perceived themselves as active, and has not been objectively measured.

### 3.3 Positive attitudes

Women who expressed positive attitudes of PA perceived themselves as active on a regular basis. They described how they managed to keep up with their exercise routines despite work and family duties, described PA in a positive manner and PA was a part of their daily life. Regular PA was expressed as a way to maintain their health, new body, and stable weight as they grew older, but also to feel strong and have enough energy for daily life.

#### 3.3.1 Positive image of PA

These women presented a variety of intensity levels of the performed activities ranging from walking to running, yoga to Zumba and training at home or going to the gym. Some described that they varied their activities depending on mood or season, while others kept to just one predominant activity. The women defined what they considered normal levels of PA, which meant an activity that you performed 2–3 times a week and that made you sweat. Additionally, they seemed to have made conscious decisions to increase their everyday PA after surgery, like biking to work, always taking the stairs, and one woman described that she had changed to a more physically demanding job. To be active on a regular basis was after surgery an important part of their lives and a routine that made them feel good. Some women had been active and exercised before their surgery too, even though it was a bit harder due to their excess weight, while others had increased their PA over time since surgery.

#### 3.3.2 Challenge myself to improve

To challenge oneself to physically improve was commonly described among the women who perceived themselves as active, and they further expressed pride in having developed a strong and capable body. They all described different ways to challenge themselves to improve their fitness and perform better, such as the use of apps to track their activities and monitor increased running distance or to keep track of increased weights lifted.

“It was never an option (before the surgery) that I would go for a run. No. No. And it is still not something I find amusing, while at the same time it is—because I feel like “yeah I can do this! I can run 10 km”. (…) And it feels good to feel like “yes, I can run up that hill three times now, next time I will try to run up four times”, so that you are challenging yourself a little.”. (Participant 8)

#### 3.3.3 To feel healthier and happier

At least one accomplished physical or mental positive aspect of regular PA was mentioned. PA was associated with joy, and the women expressed that they felt more energized, happier and slept better due to their PA. Some mentioned that they exercised regularly in order to manage physically demanding jobs better or to maintain their health as they grew older. Also, being physically active had made them more comfortable with their “new body” and they experienced more energy to accomplish daily household activities. Furthermore, exercising was a tool for being able to prevent a possible weight gain. Exercising on their own was sometimes mentioned as valuable “me-time”, as they could, for example, listen to an audiobook while performing the activity.

“And I’ve thought that it (exercising) felt good, to physically make an effort and get tired in that sort of way, it is a different kind of tiredness. I am really chatty and energized when I get home, and my family notice when I have been away (to the gym), because I get all “wiiie” like that, because I have a different energy in my body.” (Participant no. 5)

#### 3.3.4 Social support to be active

To have friends or family members that supported the women to exercise was an important aspect of start and maintaining with PA. As an example, one woman told that her husband could cook dinner while she went for a run. Also, walking the dog was a way of getting daily PA. To have support could also mean that the women exercised together with friends or family members, and this was found important as someone could push them if their motivation occasionally was lacking. Group fitness classes were another way of getting support to be active, as the class leader told the group what they should do.

“I can go by myself if it’s group (fitness) classes (…) But I think it’s fun to go with someone, especially because it’s easier to go with someone, because it may be like “I had thought of going (exercising) today but no, I am tired” and then, you know, you pick up your phone and (someone says) “shall we go (exercising) today?” And then, well I go. Because I got company.” (Participant no. 4)

#### 3.3.5 Develop strategies to remain active

The women revealed how they had developed different strategies to maintain PA in their daily lives. The women prioritized PA, and therefore they made sure to find an activity and time during the day that worked with their routines and family members, even if only short activities were possible. They also appeared to be knowledgeable about PA, or if not, they asked a personal trainer at the gym or searched the internet in order to get the knowledge they were missing. Some mentioned having loose skin but had found solutions so that they could remain active, such as wearing tight exercise clothes underneath more loose clothes.

“I think that everyone has 15 min a day (to exercise). (…) It’s just a bad excuse otherwise because 15 min a day actually exists.” (Participant no. 8)

### 3.4 Shifting attitudes

This category comprised one property, *“Constantly on and off, with some support”*, that symbolizes how some of the women continuously moved between episodes of activity and exercise, and episodes of inactivity and never managed to obtain a sustainable PA-habit. These women seemed to be constantly moving between the categories *“Positive attitudes”* and *“Negative attitudes”*, and thus identified themselves with the associated properties to either the *“positive-”* or *“negative attitude”*-category depending on which attitude they had at the moment.

#### 3.4.1 Constantly on and off, with some support

Some of the women exclaimed that they were very active in periods ranging from a few months up to half a year, followed by periods when they lost their motivation and consequently were inactive until the next episode of PA. The reasons to why the motivation of PA suddenly dropped were predominantly related to too much exercise, sometimes up to two workouts a day, which affected other responsibilities and duties. Thus, PA never became a sustainable habit long term. Some regarded themselves as “on and off-persons”, and during the “on-period” they thought the more exercise the better, but they could not explain what triggered their vigorous exercise periods. Some mentioned how they tried to get rid of the extra kilos by being more active but struggled to get some regularity and maintenance of their activities. Regarding social support, the women belonging to the group with shifting attitudes expressed that they felt supported, mostly from their spouses, to engage in PA. At the same time, they mentioned that they would have preferred more “hands on” support, i.e., having someone who could join them at the gym and exercise with together, and not only the oral support. As these women seemed to constantly be jumping between the positive and negative attitudes, they therefore also felt both supported and not supported, depending on the attitude at the moment.

“I want to find this continuity in my exercise, to not exercise really hard for half a year and then become totally dulled.” (Participant no. 5)

### 3.5 Negative attitudes

The category *“Negative attitudes”* illustrate the women who had negative attitudes towards PA and perceived themselves as quite inactive and their reasons for not exercising on a regular basis. These women expressed that they preferred a more sedentary lifestyle and did not really see any benefits of being more active. Neither did they have family or friends who supported engagement in PA.

#### 3.5.1 Negative image of PA

Most of the inactive women used words like “lazy” or ‘couch potatoes’ when describing themselves and their PA habits. Most of them stated that they were not physically limited to be active, they just didn’t like to exercise. Instead, they preferred to come home after work and engage in activities that didn’t involve PA. They viewed PA as a boring task which they lacked motivation for, and external aspects like bad weather could influence an occasional planned activity in a negative way. Some were very outspoken and said that they would never become a person who exercise regularly but described themselves as feeling strong and healthy anyway. One woman could not mention any positive aspects of PA, while another thought that PA was not important as long as you felt healthy. Some of these women had never been active, while others had been active in their youths but not since. They also mentioned that any attempts of PA had only been physically hard and too demanding before their surgery. Furthermore, some mentioned that they had no knowledge about PA, so they did not know how to get started with an activity.

“I’m probably a bit lazy in general, I am comfortable, I think. (…) I’m not a TV-person, but I can still sit like that, I can sit down and listen to my audio book for example. I haven’t watched TV that much (…) but I still sit on the couch and think it’s nice to just sit there.” (Participant no. 6)

#### 3.5.2 Priorities and wishes

For some of the women who perceived themselves as inactive, PA was not a priority. The children’s activities, family duties and work schedules were the highest priority, and some discussed that their time for PA would come later when their children grew older. Even if PA was not prioritized, some wished they were more physically active. Some talked about how they planned to start exercising later, for example, after the summer or when they started their new job or other similar life-events. Others had lost their previous habits after their near-by gym or public swimming pool had closed and had not initiated any new activity.

“But the problem is that the kids have so many activities, and we have to drive them because they can’t go there by themselves. So, it’s their activities that comes first, so if there’s something (an activity) I like to do on the day I can, then maybe it would work. But it’s not a priority for me.” (Participant no. 7)

#### 3.5.3 No social support to be active

The self-perceived inactive women with negative attitudes often described that no one in their surroundings could support them to be more active, nor did their partners or friends motivate or support them, and no one in their social environment exercised. They expressed a wish to have an exercising partner or a friend that could push them and accompany them to PA.

“No one pushes me (to exercise), no I cannot say that. (…) But yes, that (having an exercise friend) might not be such a bad thing after all. To go to the gym with someone, that’s an idea. Me and my sister have talked about it a few times, but we don’t live close to each other and she has her life and has even less free-time than I do, so…” (Participant no. 6)

#### 3.5.4 Physical limitations for PA

Physical limitations were barriers for being active for a few participants, either in terms of permanent limitations due to joint pain, or temporary injuries like a broken foot. Some had different illnesses that caused pain or tiredness, which reduced their capacity to only manage their work and daily domestic tasks. Having trouble with loose skin was also limiting, and even though many exclaimed that they were not physically limited by it, the loose skin caused psychological distress. To a few participants, the loose skin had caused so much physical troubles that they underwent body contouring surgery.

“I have done (body contouring) surgery on my stomach and chest. And they have also removed a little here on my back, so I have had surgery all around… because it was a problem, yes. And I still have problems just around my hips, so I don’t think I can run because of that. Because I think that would hurt, actually.” (Participant no. 1)

#### 3.5.5 Exercise is only equal to dieting

Almost all the women, but especially those who belonged the category “*negative attitudes*”, spoke about PA as a mean to lose weight. They described how they had previously exercised only to lose weight, and as they now had lost weight due to the surgery, they didn’t really see a point in exercising. Thus, PA was regarded as mainly an activity to achieve weight loss and an activity predominantly for fit people.

“And then I felt fat, then I didn’t have the energy. Then I felt even fatter, and then I didn’t want to go there (to the gym) because I thought that everyone that exercised were so slim, so I didn’t understand what they were doing there. It seems pointless to me to go there.” (Participant no. 4)

## 4. Discussion and conclusion

### 4.1 Discussion of the results

This study aimed to explore how women perceive and experience PA five years after RYGB surgery. The findings indicate that women’s PA was influenced by their own attitudes towards PA and whether they had a “PA-friendly” environment. Three PA attitudes were expressed; a positive attitude where women were physically active on a regular basis and felt supported and proud of their achievements, and a negative attitude where PA was not prioritized nor supported, and the women did not see the need or benefits of PA. Furthermore, a subgroup of participants showed an on-off PA-behaviour hovering between vigorous PA and a sedentary lifestyle but never finding a sustainable balance. To our best knowledge, this sub-group of patients with shifting attitudes has not previously been identified. Furthermore, in the previous qualitative studies, positive or negative attitudes towards PA have only been mentioned briefly as one of several barriers or facilitators to become physically active post-surgery (Dikareva et al., 2016), but barriers to PA are mostly categorized as different internal or external factors (Peacock, Sloan, & Cripps, 2014; Wiklund, Olbers, & Willén, 2014). Patients’ attitudes have not been designated as the main reason why most patients don’t manage to become sufficiently active after surgery, which could be a new aspect to consider when planning the post-surgery supportive care.

We found that a supportive environment seemed to be of importance if PA post-surgery would be performed regularly. These findings are in accordance with previous systematic reviews exploring the importance of social support for being physically active in all age groups (Lindsay Smith, Banting, Eime, O’Sullivan, & van Uffelen, 2017; Mendonca, Cheng, & Melo, 2014; Wendel-Vos, Droomers, Kremers, Brug, & van Lenthe, 2007). One review found a positive association between having social support, like having an exercise companion, and PA for both men and women (Wendel-Vos et al., 2007). Another review found a positive association in older adults between social support, especially from family remembers, and being physically active (Lindsay Smith et al., 2017). For adolescents, the more social support from family members and friends they received, the more PA they performed (Mendonca et al., 2014). As previously mentioned, other qualitative interview studies have also shown that lack of social support is a barrier for being physically active after bariatric surgery (Dikareva et al., 2016; Stenmark Tullberg et al., 2017; Zabatiero et al., 2017).

Another interesting finding in this study is that many of the women, especially the women who had negative attitudes about PA, thought of PA only as a mean to lose weight and not as a way of achieving other health benefits. Even though most participants mentioned positive aspects of being physically active that weren’t related to weight loss, they constantly during the interviews highlighted the aspect that PA is good and useful when you want to lose weight. Also, as the women in this study proclaimed, the motivation for exercising derived from a wish for weight loss and therefore their motivation for being physically active was lost after surgery. Similar findings have been shown in other qualitative studies. One of these studies could show that bariatric patients didn’t think it was necessary to exercise the first 6 months after surgery because they lost weight anyway (Zabatiero et al., 2017). Another qualitative study with bariatric patients post-surgery showed that a motivator for becoming (and maintaining) physically active was to lose weight or maintaining the weight-loss (Dikareva et al., 2016). Other subgroups have displayed similar results. One study interviewed obese individuals that participated in an exercise program at a dietetic clinic about their experiences of PA (Guess, 2012). The result shows that the participants could mention several non-weight related health-benefits with PA, but weight loss was still the primary motivation for exercising. These findings are in accordance with the finding in our study. If most bariatric patients only view PA as a mean to lose weight, it is understandable why this patient group fails to meet the MVPA guidelines, as they don’t see the point of exercising or to be physically active because of the surgery-induced weight loss. These findings highlight the need for educational interventions to motivate sustainable PA as part of the post-surgery support as to improve health and well-being over time.

The explanatory model of the findings can be discussed using cognitive dissonance-based theory (Stone & Focella, 2011), a theory based on the assumption that the mind can’t hold two inconsistent cognitions at the same time, or psychological distress occurs. For example, you want to exercise regularly but you don’t, resulting in distress and discomfort; which can be the motivation for a change of behaviour so that the behaviour fits the cognition of being a person that exercise. Applying a dissonance-based theory may explain the perceived PA-behaviours revealed in this study; some women had positive attitudes towards PA post-surgery and therefore managed to develop strategies to overcome any psychological distress that might occur if not being active. On the other hand, the women with negative attitudes had their reasons and explanations (e.g., children’s activities or work schedule) to remain sedentary, thus no psychological distress appeared that made them motivated to increase their PA.

Besides the general health benefits of PA, PA among bariatric surgery patients has shown to improve surgical outcomes (Jacobi et al., 2011; Wc & Ds, 2013; Wefers et al., 2016). We found that some patients do not see the need of PA after bariatric surgery. Therefore, there is a challenge for health-care providers encountering patients after bariatric surgery to provide information, support and to emphasize all the positive benefits with PA, so that bariatric patients can find the right attitude and motivation for sustainable PA. For future research, it would be beneficial with interventions investigating if bariatric surgery patients’ attitudes towards PA can be influenced in a positive direction, and in conjunction, also investigate if a more positive attitude would objectively increase patients PA post-surgery. A tool to capture the patients with negative attitudes could be developed to be used by health-care providers, in order to give the patients the additional support they need to become physically active after bariatric surgery.

### 4.2 Methodological considerations

There are some limitations in this study that need to be addressed. A final sample of 11 out of 38 eligible women volunteered to be interviewed. The sample of participants may have been biased as only the most talkative and active women may have agreed to be interviewed. Also, previous research has shown that the majority of patients after bariatric surgery is not sufficiently active (Bergh et al., 2017; Berglind et al., 2015, 2016; Bond et al., 2010; Sellberg et al., 2017), so eligible participants may be reluctant to reveal their experiences; thus, choose not to volunteer. Patients who have undergone RYGB surgery have previously belonged to a stigmatized and discriminated population (Puhl & Heuer, 2010), so there may be biased reporting by participants in order to make themselves look more favourable. Also, in this study we only present their self-reported view of themselves regarding PA, and from previous research we know that self-reported PA does not reflect objective measured PA (Bergh et al., 2017; Berglind et al., 2015, 2016; Bond et al., 2010; Sellberg et al., 2017). Despite the possible biases, the participants were outspoken and frank about their experiences irrespective of perceived activity level, and shared personal and intimate information about their post-surgery lives; facts that may strengthen the trustworthiness of these findings. A strength of this study is the great variation of expressed experiences and perceptions concerning PA. The last three interviews did not bring any new essential information into the analysis; therefore, saturation of data was most likely established. To increase the transferability of the result, rich descriptions of the procedure and analysis were provided. Also, measures were taken during the research process to secure trustworthiness and reduce biases to the result. To ensure dependability in this study, an audit trial of memos was used during the whole process as well as a clear coding strategy that was easily traceable and transparent. Additionally, all interviews were performed by the same researcher which minimize biased interview procedures. In order to achieve conformability, researchers with different backgrounds were involved in the analysis and discussion of the results as to reduce biased interpretation of the analysis. Furthermore, to enhance credibility the result was discussed and confirmed through peer-reviewing with research colleagues outside of the research group.

Even if PA may not seem like a sensitive topic to address, any topic discussed during interviews has the potential of being sensitive (Corbin & Morse, 2003), and therefore it is of importance to consider the potential risks and biases (Corbin & Morse, 2003; Dempsey, Dowling, Larkin, & Murphy, 2016). However, none of the participants seemed stressed or upset during the interviews and without indications of perceived unpleasant experiences or long-term distress after the interviews.

We asked the participants about their views of their PA-behaviour, and not specifically their MVPA because we wanted to include the perceptions and experiences of all their physical movements. Thus, when asking about a participants’ PA, we will also indirectly receive information about their perceptions and experiences of their perceived MVPA, as the women often used the word “exercise” when describing their activities.

In conclusion, the result suggests that women’s attitudes towards PA and whether they had a “PA-friendly” environment influenced their self-perceived PA-behaviour five years after RYGB-surgery. Furthermore, to our best knowledge, a subgroup not previously identified showed an on-off PA behaviour hovering between vigorous PA and sedentary lifestyle, but never finding a sustainable balance together with little support. These findings highlight the need for prolonged support and motivational interventions to promote sustainable PA after bariatric surgery. A major challenge for the post-surgery supportive health care is to educate patients about benefits of PA and help over bridging patients’ PA barriers so that all patients can obtain the benefits of being sufficiently physically active after bariatric surgery.

## Data Availability

The data sets used and analysed during the current study are available from the corresponding author on reasonable request.
